# Strawberry fruit FanCXE1 carboxylesterase is involved in the catabolism of volatile esters during the ripening process

**DOI:** 10.1093/hr/uhac095

**Published:** 2022-04-22

**Authors:** Félix Juan Martínez-Rivas, Rosario Blanco-Portales, Enriqueta Moyano, Saleh Alseekh, Jose Luis Caballero, Wilfried Schwab, Alisdair R Fernie, Juan Muñoz-Blanco, Francisco Javier Molina-Hidalgo

**Affiliations:** Department of Biochemistry and Molecular Biology, University of Córdoba, Edificio Severo Ochoa, Campus de Rabanales, E-14014 Córdoba. Spain; Max Planck Institute of Molecular Plant Physiology, Am Mühlenberg 1, 14476 Potsdam-Golm, Germany; Center for Plant Systems Biology and Biotechnology, 4000 Plovdiv, Bulgaria; Department of Biochemistry and Molecular Biology, University of Córdoba, Edificio Severo Ochoa, Campus de Rabanales, E-14014 Córdoba. Spain; Department of Biochemistry and Molecular Biology, University of Córdoba, Edificio Severo Ochoa, Campus de Rabanales, E-14014 Córdoba. Spain; Max Planck Institute of Molecular Plant Physiology, Am Mühlenberg 1, 14476 Potsdam-Golm, Germany; Center for Plant Systems Biology and Biotechnology, 4000 Plovdiv, Bulgaria; Department of Biochemistry and Molecular Biology, University of Córdoba, Edificio Severo Ochoa, Campus de Rabanales, E-14014 Córdoba. Spain; Biotechnology of Natural Products, Technische Universität München, Liesel-Beckmann-Str. 1, 85354 Freising, Germany; Department of Biochemistry and Molecular Biology, University of Córdoba, Edificio Severo Ochoa, Campus de Rabanales, E-14014 Córdoba. Spain; Department of Biochemistry and Molecular Biology, University of Córdoba, Edificio Severo Ochoa, Campus de Rabanales, E-14014 Córdoba. Spain; Department of Biochemistry and Molecular Biology, University of Córdoba, Edificio Severo Ochoa, Campus de Rabanales, E-14014 Córdoba. Spain

## Abstract

Volatile compounds produced during ripening of strawberry are key determinants of fruit quality and consumer preference. Strawberry volatiles are largely esters which are synthesized by alcohol acyltransferases (AATs) and degraded by carboxylesterases (CXEs). Although CXE activity can have a marked influence on volatile contents in ripe strawberry fruits, CXE function and regulation in them are poorly known. Here, we report the biochemical and functional characterization of the fruit receptacle-specific and ripening-related carboxylesterase FanCXE1. The expression of the corresponding gene was found to be antagonistically regulated by auxins and abscisic acid, key hormones that regulate fruit growth and ripening in strawberry. *In vitro*, FanCXE1 was able to hydrolyze artificial ester substrates similar to those produced by ripe strawberry fruits. Transient suppression of the *FanCXE1* gene by RNAi resulted in an increase of important volatile esters such as methyl hexanoate, methyl butanoate and ethyl hexanoate as well as a decrease of the alcohols hexenol and linanool. The results of this work enhance our understanding of the molecular basis for volatile syntheses and facilitate production of better flavored strawberry fruits by introduction of the relevant alleles into common cultivars.

## Introduction

The unique flavor of strawberry is due to a complex mixture of sugars, acids and volatile compounds whose contents and balance define the fruit taste. The sweet compounds are mainly sugars in the form of glucose, fructose and sucrose, whereas those determining acidity are organic acids (citric and malic, mainly). [1] Strawberry fruits additionally contain more than 900 volatiles including esters, alcohols, ketones, furans, terpenes, aldehydes and sulfur compounds. [2] Although these volatiles are present at very low levels (typically 0.001–0.01% in fresh weight), minor changes in their composition can dramatically alter strawberry taste. [3, 4]

The odor activity value (OAV) of a volatile compound is a measure of its contribution to aroma perception. [4, 5] Based on OAVs, the greatest contributors to strawberry fruit aroma are ethyl butanoate, ethyl hexanoate, methyl butanoate and methyl hexanoate among esters; 2,5-dimethyl-4-hydroxy-3(2H)-furanone (DMHF) and 4-methoxy-2,5-dimethyl-3(2H)-furanone (DMMF) among furanones; linalool and nerolidol among terpenes; and methanethiol among sulfur compounds. [5] However, volatiles are not only related to aroma, but also to plant defense mechanisms against herbivores and pathogens, plant-to-plant interactions and attraction of pollinator disperse seeds. [6]

Carbohydrates, fatty acids and amino acids are known to be the main precursors of most aroma related volatiles, which include straight-chain aldehydes, lactones, ketones, alcohols and esters. [7] The main contributors to fruity aroma are esters such as methyl butanoate, ethyl butanoate, butyl butanoate, methyl hexanoate, ethyl hexanoate, butyl acetate and hexyl acetate. [8] Furans (specifically, furaneol and mesifurane) add caramel notes, while the terpenoids linalool and nerolidol impart flowery notes. The so-called “green volatile compounds” (*Z*)-3-hexenal, (*E*)-2-hexenal and (*Z*)-3-hexen-1-ol contribute to traits that typically decrease with ripening. Finally, γ-decalactone, another major volatile, confers “peach-like” notes. [8, 9] The previous compounds are mainly produced through the lipoxygenase pathway, where hexanal and (*Z*)-3-hexenal are synthesized from linoleic (18:2) and linolenic (18:3) acid, respectively. The resulting aldehydes are converted into alcohols under catalysis by alcohol dehydrogenase (ADH). At the end of the process, alcohol acyl transferases form an ester that catalyzes a reaction between the acyl moiety and the alcohol residue —one that can be reversed by carboxylesterases (CXEs). In fact, in the presence of water, these enzymes catalyze the hydrolysis of carboxylic esters into alcohols and carboxylic acids.

Fatty acids can also be degraded by α- and β-oxidation in the presence of two AAT enzymes with high sequence homology but different substrate affinity. [10, 11] Also, γ-decalactone production is known to be related to the expression of the omega-6 fatty acid desaturase FaFAD, [9] and the enzyme *O*-methyltransferase (FaOMT) to convert furaneol into mesifurane. [12] Moreover, specific alleles of *nerolidol synthase1 (*FaNES1) and *(−)-*α*-pinene synthase* (FvPINS) have been associated with production of the monoterpenoid linalool and the sesquiterpenoid nerolidol in the former case, and of the monoterpene α-pinene in the latter. [13]

Plant carboxylesterases (CXE, EC 3.1.1.1) are hydrolytic enzymes belonging to the α/β hydrolase fold superfamily of proteins. CXEs have a conserved catalytic triad consisting of a serine residue contained in the conserved sequence GXSXG, an acidic amino acid, and a histidine residue that constitute their active site [14–16]. The specific functions of CXEs in plants are poorly understood. They might be associated to major biochemical and physiological functions including degradation of xenobiotics and conversion of volatiles that determine the aroma of ripe fruits. [17–20] CXE enzymes have also been related to biotic stresses. Thus, AtCXE8 in *Arabidopsis thaliana* is one agent of the plant’s response to the fungal pathogen *Botrytis cinera*. [21] Also, the resistance of transgenic pepper fruits by overexpressing the *PepESTs* gene in its interaction with the fungal pathogen *Colletotrichum gloesporoides* is at least partially mediated by an increased amount of the encoded CXE enzyme (PepEST). [22]

The functional role of CXEs in fruit ripening has scarcely been examined. The ripening-related carboxylesterase MdCXE1 in *Malus* is thought to be indirectly associated with the hydrolysis of flavor esters to butyl and hexyl acetates, which are key components of the ripe fruit aroma. [17] Similarly, SlCXE1 has been deemed a regulator of the contents in volatile acetate esters in tomato (*Solanum lycopersicum*). [23] Recently, a functional analysis of 33 CXEs in peach revealed that 13 were expressed in fruits; that expression of 6 of those 13 members (viz., *PpCXE*1, *PpCXE*2, *PpCXE*3, *PpCXE6, PpCXE*27 and *PpCXE*32) was ripening-related; and that the *PpCXE*1, *PpCXE*2 and *PpCXE*3 transcripts were most abundant in ripe fruits. Functional enzymatic and genetic studies have shown the enzymes behind these three genes to hydrolyze volatile esters during ripening. [18, 19]

Transcriptomic analysis previously allowed our group [24] to identify a gene with receptacle-specific and ripening-related expression. The gene, named *FanCXE1*, exhibited considerable sequence homology with carboxylesterases from higher plants such as apple, peach and tomato. In this work, we addressed its functional characterization. Based on the results, FanCXE1, like PpCXE1, can hydrolyze esters both *in vitro* and *in vivo.* Transient downregulation of *FanCXE1* expression in fruit receptacles increased the levels of esters such as methyl hexanoate, methyl butanoate and ethyl hexanoate, and also those of the alcohols hexenol and linanool. Therefore, FanCXE1 influences production of volatiles during ripening in strawberry fruit.

## Results

### Bioinformatic and phylogenetic analysis of the *FanCXE1* gene and protein

Up to 33 (52 isoforms) and 75 (110 isoforms) putative CXEs have been annotated in the latest version of *F. vesca* and *F. × ananassa* genome, respectively. [25, 26] Transcriptomic analysis previously revealed FanCXE1 to have a ripening-related expression pattern. [24] As shown here, it is strongly upregulated during ripening. The gene has a 981 bp open reading frame. Also, its protein sequence has signatures similar to those of proteins in the CXE family including the transmembrane region of the α/β hydrolase fold in the superfamily transmembrane region (see Supplementary Fig. S1). In addition, as revealed by a BLASTP search, there are close similarities between *FanCXE1* and carboxylesterases from other higher plants, identities ranging from 50% to 78%.

In this work, we conducted a phylogenetic analysis of FanCXE1, the 52 putative CXE isoforms of *F. vesca*, the 110 putative isoforms of *F. × ananassa*, and a further 16 CXEs from apple, [17] 20 from *A. thaliana* [14] and 32 from peach [18] (Supplementary Fig. S2). FanCXE1 has been related to MdCXE8 in apple, and to the isoflavanone dehydratases HIDM and HIDH in soybean and licorice, which belong to Arabidopsis clade III. [17] FanCXE1 has similar physicochemical properties, isoelectric point (pI), molecular weight (Mw) and parallel prediction of subcellular loci (Supplementary Table S1). The predicted catalytic triad (Ser, Acid and His), including the pentapeptide sequence (GXSXG) surrounding the nucleophilic serine, is conserved in all CXEs and also present in the inferred amino acid sequence of FanCXE1 (Supplementary Fig. S3). The serine residue at the FanCXE1 active site is present in almost all other carboxylesterase-like proteins such as MdCXE8, PpCXE12 and PpCXE13. FanCXE1 additionally contains a conserved Gly-82-Gly-83 sequence that forms an oxyanion hole within the carboxylesterase motif —the typical motif of CXEs [27] (Supplementary Fig. S3).

### Spatio–temporal and hormonal expression profile of the *FanCXE1* gene

**Figure 1:**
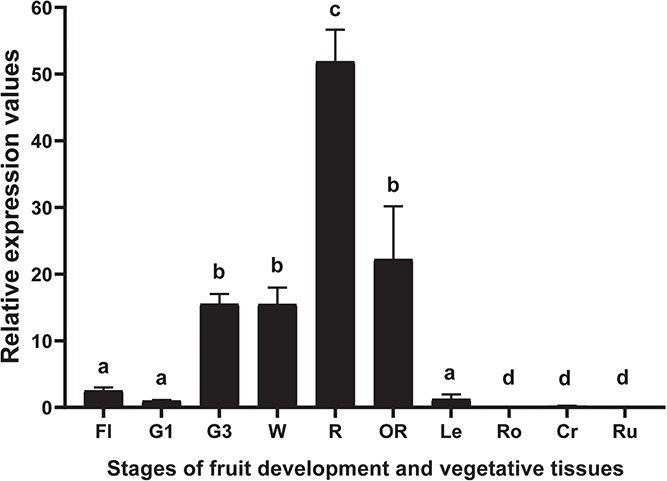
Expression analysis of *FanCXE1* in strawberry fruit receptacles and in vegetative tissues of *F.* × *ananassa* cv. Camarosa. Expression in receptacle G1 was used as reference and given an arbitrary value of 1. Values are means ± SD for five independent experiments. Fl flower, G1 small-sized green fruit, G3 full-sized green fruit, W white stage, R red stage, OR overripe stage, Le leaf, Ro root, Cr crown, Ru runner. Statistical significance was assessed by one-way ANOVA. Letters indicate significant differences at *p* < 0.05 as per Scheffe’s post-hoc test.

*FanCXE1* is low in transcript abundance in flowers, and during the developmental and elongation stages in fruits. It increases throughout ripening and peaks at the red ripe stage (Fig. 1); also, it is preferentially expressed in fruits and minimally expressed in vegetative tissues (Fig. 1).

**Figure 2:**
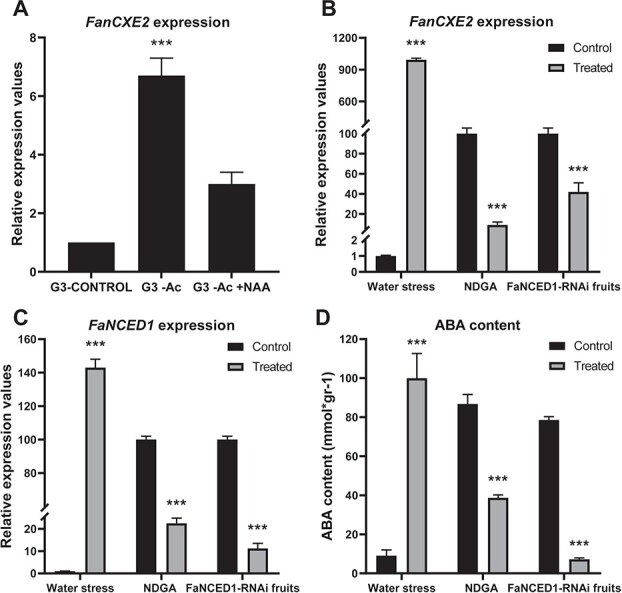
*FanCXE1* gene expression in response to hormone treatments. (A) Auxin treatments. *FanCXE1* expression was determined relative to expression in G3 fruits (control), which was assigned an arbitrary value of 1. G3-Control middle-sized green fruit receptacle. G3-Achenes G3 fruit receptacle without achenes for 5 days. G3-Achenes+NAA G3 fruit receptacle without achenes plus NAA for 5 days (added at day zero). (B) Analysis by qRT-PCR of *FanCXE1* gene expression in strawberry G-W fruits under water stress, strawberry G-W fruits treated with NDGA and G-W fruits agroinfiltrated with the NCED1-RNAi construct. (C) Analysis by qRT-PCR of *FaNCED1* gene expression in strawberry G-W fruits under water stress, strawberry G-W fruits treated with NDGA and G-W fruits agroinfiltrated with the NCED1-RNAi construct. (D) Determination of ABA content in strawberry G-W fruits under water stress, strawberry G-W fruits treated with NDGA and G-W fruits agroinfiltrated with the NCED1-RNAi construct. Statistical significance with respect to the reference sample was determined with Student’s *t*-test. (***) *p* ˂ 0.001.

Ripening in strawberry fruit receptacles is triggered by a certain ABA/auxin ratio. [24, 28] Auxins, which are produced mainly in achenes, are released to receptacles, where they promote growth and development while avoiding premature ripening. In this work, the *in vivo* effect of auxins was examined by comparing *FanCXE1* expression in untreated and auxin-treated de-achened G3 fruits. *FanCXE1* was more markedly expressed in de-achened fruits (Fig. 2A). Its expression, however, was scarcely induced by external auxin. The influence of ABA on *FanCXE1* expression was assessed by following three well-validated experimental approaches, namely [29]: (*a*) depleting water in the fruit —drought stress is known to increase ABA contents in plants—; (*b*) knocking down expression of *FaNCED1*, which codes 9-*cis*-epoxycarotenoid dioxygenase —the key enzyme in ABA biosynthesis—; [30] and (*c*) adding NDGA, an inhibitor of NCED1 activity (Fig. 2B). The three types of test were accompanied by assessment of the relative expression of *FaNCED1* as a measure of ABA biosynthesis (Fig. 2C) and by the determination of ABA in fruit receptacles (Fig. 2D). ABA contents were markedly increased by water stress conditions but considerably decreased in fruit receptacles treated with NDGA or in which *FaNECD1* expression had been silenced. Hormonal studies previously showed *FanCXE1*, like other strawberry ripening-related genes, to be regulated antagonistically by ABA and auxin. [25].

### Subcellular location of FanCXE1 protein.

**Figure 3:**
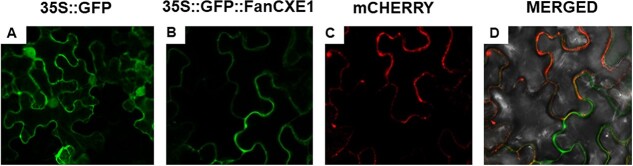
Subcellular loci of FanCXE1 protein. *Nicotiana benthamiana* leaves were agroinfiltrated with translational constructs 35S::GFP as controls (A), 35S::GFP::FanCXE1 (B) and plasma membrane mCherryFP marker (pm-rk CD3–1007, Nelson & Nebenführ., 2007) (C). Merged image of GFP fluorescence, mCherryFP fluorescence and brightfield (D).

Bioinformatic analysis of the amino acid sequence in FanCXE1 protein suggested the presence of a single putative transmembrane region (Supplementary Fig. S1). The assumption that FanCXE1 protein was anchored to the plasma membrane in plant cells was confirmed by translational fusion of FanCXE1 and GFP. For this purpose, *Agrobacterium tumefaciens* carrying either 35S::GFP::FanCXE1, a plasma membrane marker (PM) tagged to mCherryFP, or the control construct 35S::GFP, was infiltrated into *Nicotiana benthamiana* leaf cells. As expected, the 35S::GFP construct was present in the whole cell (Fig. 3A), whereas GFP::FanCXE1 fusion protein was targeted to plasma membranes (see the overlay projections of confocal stacks in Figs 3B–3D, where colocalization is clearly apparent). These results suggest that FanCXE1 protein is linked to the plasma membrane.

### Enzymatic characterization and catalytic properties of FanCXE1 recombinant protein

Enzymatic activity in FanCXE1 was assessed by semipurifying an induced FanCXE1-GST *E.coli* extract with GST-tag resin. The extract contained a protein of Mw = 64 kDa (viz., 37 kDa for FanCXE1 and 27 kDa for the GST-tag; Supplementary Fig. S4). FanCXE1 was characterized kinetically *in vitro* by using α-naphthyl acetate, α-naphthyl butyrate and β-naphthyl hexanoate as artificial substrates (Table 1).

### Influence of time, enzyme concentration and substrate specificity

**Figure 4:**
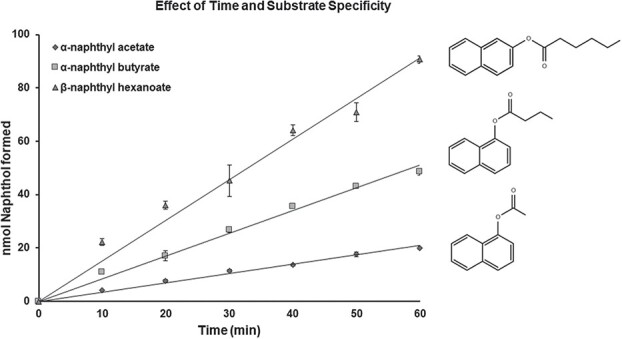
Effect of time and substrate specificity of strawberry recombinant protein, FanCXE1. Conditions: 0.3 mM substrate, 0.05 M sodium phosphate buffer, pH 7.0, 27°C. Nonenzymatic hydrolysis of the substrate was assessed by using control samples containing GST protein and 1-naphthol formed was determined colorimetrically. Error bars represent standard errors of the means as calculated from a minimum of 3 replicates each.

FanCXE1 hydrolyzed α-naphthyl and β-naphthyl esters *in vitro*. Catalytic activity in the enzyme was assessed by examining the effect of time and the enzyme concentration on the reaction. The reaction rate increased linearly up to a an amount of 125 μg of protein and a reaction time of 60 min. FanCXE1 exhibited a specific activity of 300, 900, and 1500 pmol protein/min for α-naphthyl acetate, α-naphthyl butyrate and β-naphthyl hexanoate, respectively; also, it was especially active toward β-naphthyl hexanoate (Fig. 4).

### Influence of pH and temperature on the activity of purified esterase

To assess the optimum pH and temperature for the FanCXE1 enzyme, we tested several conditions. The optimum pH for FanCXE1 action was between 7 to 7.5 (Supplementary Fig. S5A) and the optimum temperature between 25 to 30°C (Supplementary Fig. S5B).

### K_m_ and V_max_

*K*_m_ and *V*_max_ were determined by using the software Graphpad Prism 6. The *K*_m_ values for α-naphthyl acetate, α-naphthyl butyrate and β-naphthyl hexanoate were 0.68, 1.58 and 0.57 mM, respectively, whereas their *V*_max_ values were 0.24, 0.99 and 2.16 pKat μg^−1^, respectively (Table 1).

### Transient silencing of FanCXE1 alters volatile contents

Transient knockdown of *FanCXE1* in strawberry fruits was used to further confirm the *in vivo* role of this enzyme. *FanCXE1* expression was transiently downregulated by agroinfiltrating the RNAi construct pFRN-FanCXE1 into fruits. In parallel, control fruits were agroinfiltrated with pFRN empty vector. *FanCXE1* expression was assessed by comparing the results for transgenic and control fruits (Supplementary Fig S6). Metabolites were determined in three pools comprising the most markedly silenced fruits.

**Figure 5:**
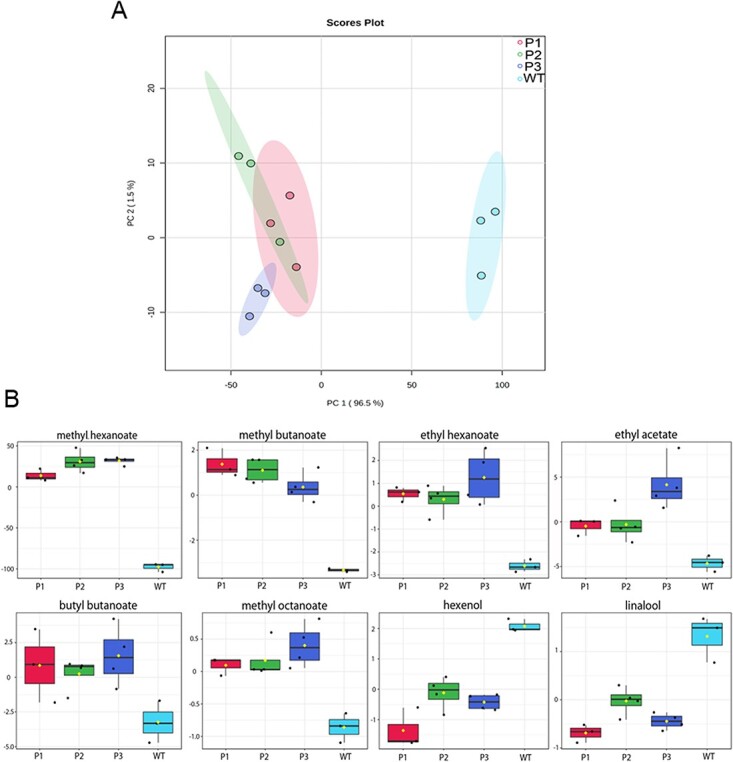
(A) Principal component analysis of metabolites identified in receptacles of control samples injected with pFRN-empty (gray dots) against three transgenic fruit pools injected with pFRN-FanCXE1 (green, blue and red dots). Three replicates were used under each set of conditions in the metabolomic analysis. (B) Relative concentrations of various esters and alcohols in transgenic and control receptacles. P1 pool 1, P2 pool 2, P3 pool 3, WT wild type fruits. Data were scaled by using Pareto’s method.

**Figure 6:**
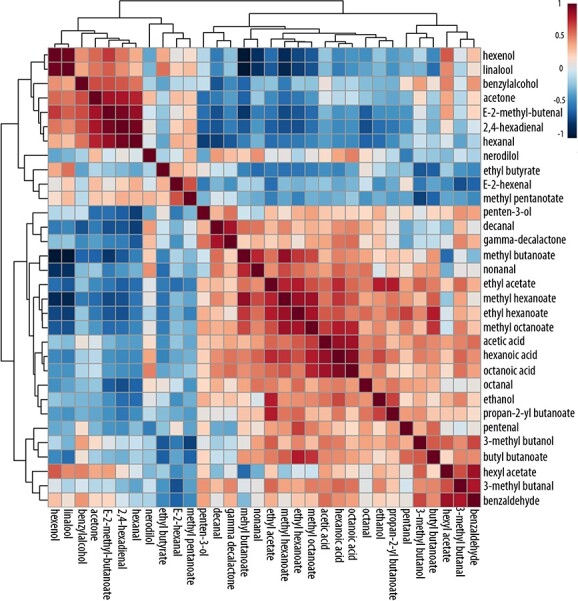
Correlation of volatile compounds identified in transgenic and control receptacles. Relative metabolite concentrations were scaled and analyzed by using Spearman’s rank correlation analysis. Each colored cell on the map indicates the correlation coefficient, with the scale code shown at the top right corner (red and blue denote positive and negative correlations, respectively).

Volatile contents were determined in infiltrated fruits. Principal component analysis (PCA) revealed marked differences in metabolite composition between control and silenced fruits (Fig. 5A), thus confirming that FanCXE1 clearly plays some role in strawberry volatile production. As far as individual esters are concerned, the levels of methyl and ethyl hexanoate, ethyl acetate, butyl butanoate, methyl octanoate and methyl butanoate were substantially increased in transgenic receptacles relative to control fruits. Also, compounds containing hydroxyl groups such as linalool and 1-hexanol were present at lower levels in transgenic fruits than they were in control fruits (Fig. 5B). By contrast, other key compounds of the strawberry volatilome such as γ-decalactone, ethanol and hexanoic acid were present at essentially identical levels in both groups. Relationships between volatiles were explored by using Pearson’s correlation analysis. Data clearly grouped into two clusters each containing compounds with similar chemical structures (viz., alcohols and esters). The contents in esters such as methyl butanoate and methyl hexanoate were inversely correlated with those in compounds containing hydroxyl groups such as nerolidol, linalool and benzyl alcohol (Fig. 6).

**Table 1:** Kinetic parameters for the hydrolysis of various naphthyl esters with semipurified recombinant FanCXE1. Standard errors of the means were calculated from a minimum of three replicates each

**Ester**	***K***_**m**_ **(mM)**	***V***_**max**_ **(pKat μg** ^**−1**^**)**	***V***_**max**_ **/*K***_**m**_ **(pKat μg** ^**−1**^ **mM**^**−1**^**)**
α-Naphthyl acetate	0.678 ± 0.17	0.24 ± 0.009	0.35
α-Naphthyl butyrate	1.579 ± 0.19	0.99 ± 0.022	0.62
β-Naphthyl hexanoate	0.572 ± 0.25	2.16 ± 0.134	3.77

## Discussion

Transcriptomic comparison of green and red fruits allowed a gene encoding a putative carboxylesterase whose expression was strongly upregulated in ripe fruits to be identified. [24] Bioinformatic analyses showed the FanCXE1 deduced protein sequence to contain well-conserved functional domains present in carboxylesterase proteins such as an α/β hydrolase fold domain, the GDXG lipolytic domain and a conserved transmembrane region (Supplementary Figs S1 and S3). This structural evidence suggests that the protein in question might possess carboxylesterase activity. The phylogenetic relationship among FanCXE1 and other plant carboxylesterases indicates that, based on CXE classification, [14] FanCXE1 belongs to clade III together with HIDH dehydratases (Supplementary Fig S2). HIDs belong to a small group of proteins from leguminous plants in clade III. FanCXE1 is in clade III together with HIDH enzymes; like MdCXE8, PpCXE12 and PpCXE13, however, it has a serine residue in the catalytic triad rather than the threonine residue present in HIDHs (Supplementary Fig. S3). Targeted mutation of Thr to Ser in soybean HIDH causes a loss of enzyme activity relative to dehydration in isoflavonones. [32]

## FanCXE1 affinity increases with increasing chain length of the naphthyl ester

Production of recombinant FanCXE1 in bacteria, and its semipurification, allowed its carboxylesterase activity to be assessed. The optimum functional pH, 7.5, is similar to those for other CXEs (7.0–9.0). [17, 19] The catalytic triad in CXEs, which contains a deprotonated histidine residue (p*K*_a_ = 6.0), requires a neutral to basic pH to trigger serine at the active site. [33] FanCXE1 exhibited a high affinity for the longer naphthyl esters and, specifically, for β-naphthyl hexanoate (Fig. 4; Table 1). These catalytic properties are similar to those of other carboxylesterases such as those present in apple [17] and peach. [19]

### *FanCXE1* is a fruit-specific gene and responds to the key hormones governing ripening

The expression pattern of *FanCXE1* was clearly related to ripening; thus, it was markedly strong at the red fruit stage. A similar pattern was previously found in *MdCXE1* from *Malus*, [17] and also in *PpCXE1*, *PpCXE2*, *PpCXE3*, *PpCXE6*, *PpCXE27* from *Prunus*. [18, 19] qPCR analysis revealed that only *SlCXE1* among the five CXEs identified in *S. lycopersicum* was highly expressed in ripe fruits. Also, *SlCXE1* expression was mainly restricted to fruits, and scarce in flowers and leaves. [23] In this respect, *SlCXE1* is very similar to *FanCXE1*, which was preferentially expressed in fruit receptacles but weakly or not at all in vegetative tissues (Fig. 1).

Because strawberry is a nonclimacteric fruit, ethylene production in it does not increase with ripening; rather, the process is triggered by a certain ABA to auxin ratio. [24, 28, 30] Changes in fruit size and shape at an early stage of development are mainly due to auxins. [34]. However, once fruits have reached full size, auxin levels fall and ripening starts. [28, 34] Many ripening related strawberry genes are prematurely induced upon removal of achenes. [10, 24, 35–37] In fact, removing achenes from G3 fruits resulted in premature induction of *FanCXE1*. However, this induction was reversed by application of auxin NAA to previously de-achened fruits, which indicates that the *FanCXE1* gene is also negatively regulated by auxins (Fig. 2A). By contrast, ABA levels increase gradually with ripening in strawberry. [30, 38] The role of ABA in *FanCXE1* expression was investigated here in three different experiments involving modulation of ABA levels in the fruit. Two were directly related with its biosynthetic pathway and involved inhibiting NCED activity or silencing expression by RNAi. Both experiments led to a decrease in ABA levels that correlated with *FanCXE1* expression levels (Fig. 2B). Thus, water stress induced ABA production in the fruits and subsequently increased *FanCXE1* transcript levels (Fig. 2B), which is consistent with a positive regulation of *FanCXE1* by ABA. Taken together, these results suggest a marked impact of hormone levels on FanCXE1 expression. This expression pattern is consistent with those of other genes influencing organoleptic properties in strawberry and taking part in the metabolism of volatiles, cell walls and phenylpropanoids [10, 36, 39, 40].

## FanCXE1 plays a role in volatile ester catabolism in strawberry fruit

*Fragaria* wild species are considerably more aromatic than modern cultivars of the genus such as *F. **×** ananassa*, [41, 42] which have been developed to improve grower-appreciated traits such as yield, firmness and fruit size. The new cultivars, however, possess a reduced volatile diversity. [43] Phenotyping volatiles is a bottleneck in the process of improving strawberry fruit flavor and aroma as a result of quantifying volatiles being expensive and time-consuming —and also, frequently, unaffordable by most breeding programs.

In most fruits —strawberry included—, volatile release starts with ripening and increases steadily ever after. Strawberry volatiles are mainly esters, which account for 25–90% of all volatiles depending on the particular cultivar. [44] The esters are synthetized by alcohol acyltransferases and broken down by carboxylesterases. The amounts of volatile esters and alcohols that are produced depend on the activity ratio between these two antagonistic enzymes Two genes encoding alcohol acyltransferases (SAAT and AAT2 [10, 11]) have been characterized in strawberry, and one each in wild *F.vesca* (VAAT [13]) and *Fragaria chiloensis* (FcAAT [45]). Both *F. **×** ananassa* enzymes are induced during ripening, their expression being downregulated by auxins. While FaAAT2 is especially active with C6–C10 alcohols and less so with short-chain alcohols, FaSAAT exhibits the opposite trend. As a result, which esters are present in the fruits depends on the availability of substrates and the specificity of AAT enzymes. [10, 11] However, no carboxylesterases have so far been characterized in strawberry, so their functional activities in this fruit are unknown. In this work, we aimed at filling this gap in knowledge by investigating the potential role of *FanCXE1* in the hydrolysis of esters, which are among the main components of the volatilome of ripe strawberry fruit.

Two fruit related carboxylesterases (viz., *Malus* MdCXE1 and *Prunus* PpCXE1) were recently characterized. Like *FanCXE1*, both are expressed during ripening; also, their expression patterns are modulated by ethylene, which is the main hormone boosting ripening in these climacteric fruits, demonstrating their role during ripening. [17, 19] Overexpression of *PpCXE1*, transiently in peach and stably in tomato, was previously found to decrease the contents in volatile esters, which suggests that this enzyme plays a key role in their regulation. [18, 19] The *in vitro* activity of the recombinant FanCXE1 protein and its spatio–temporal expression profile suggest that it may play an equally important role in volatile conversion during strawberry ripening. Transient downregulation of *FanCXE1* expression here resulted in marked differences in volatile contents between transgenic and control receptacles (Fig. 5A). Transgenic fruits accumulated greater amounts of esters (viz., methyl and ethyl hexanoate, ethyl acetate, butyl butanoate, methyl octanoate and methyl butanoate). By contrast, alcohols such as linalool and 1-hexenol were less abundant, which suggests a role of FanCXE1 in their accumulation (Fig. 5B). Based on these results, knocking down *FanCXE1* expression affects ester and alcohol contents in an opposite manner (Fig. 6). Overexpression of PpCXE1 in peach fruit was previously found to decrease the contents in volatile esters such as hexyl acetate, *E-*hexenyl acetate and *Z-*3-hexenyl acetate; [19] also silencing *SlCXE1* in tomato increased the levels of acetate esters [23] These two enzymes play a catalytic role similar to that of FanCXE1. By contrast, the levels of other volatile compounds such as γ-decalactone, ethanol and hexanoic acid exhibited no differences, thus showing that FanCXE1 activity is highly specific and restricted to a handful of esters (Fig. 6). The *in vitro* activity of FanCXE1 in hydrolyzing esters, and the increased ester contents resulting from *FanCXE1* knockdown *in vivo*, clearly indicate that this enzyme catalyzes an important step of volatile ester catabolism in strawberry. Indeed, FanCXE1 seemingly contributes considerably to raising alcohol levels by hydrolyzing increasingly abundant esters during ripening.

## FanCXE1 is linked to cell membranes

Carboxylesterases in plants are involved in various biological processes and can be allocated to different subcellular components. [19] Our experiments aimed at identifying the subcellular localization of FanCXE1 were done with GFP fusion protein. Although FanCXE1 was targeted in the cell membrane (Fig. 3), alternative subcellular locations such as the cytoplasm or endoplasmic reticulum cannot be ruled out. This is consistent with the predicted localization obtained in different by some predictor servers and similar to that for alcohol acyltransferases, which suggests that the concomitance of biosynthetic and hydrolytic enzymes may start a cycle between esters and alcohols leading to modulation of the volatile composition. [46, 47] However, since little is known concerning the subcellular localization of other enzymes, and of their relationship with the molecular mechanisms of these enzymes and their action in fruits, still makes it difficult to assess the physiological significance of their subcellular distribution.

## Conclusions

Carboxylesterases (CXEs) constitute a large, diverse, complex group of enzymes with overlapping substrate specificities. Very little is known about their natural substrates, however. As shown here, FanCXE1 contributes to the production of alcohols by hydrolysis of esters during ripening. Overall, these results suggest that FanCXE1 plays a role in ester metabolism in ripe strawberries by altering their ester profile. This finding can improve our understanding of the molecular basis for fruit volatiles and allow superior alleles of relevant genes to be introduced into common cultivars with a view to obtaining better flavored fruits. Such products can be expected to be favored by consumers, and hence to promote healthier eating habits and increase profitability for strawberry growers.

## Materials and methods

### Plant material

The strawberry plants studied (*Fragaria × ananassa* Duch. cv. Camarosa, an octoploid cultivar) were grown under field conditions in Huelva (SW Spain). The fruits were harvested at various developmental stages and classified as follows: small-sized green fruits (G1, 2–3 g), full-sized green fruits (G3, 4–7 g), white fruits (W, 5–8 g), full-ripe red fruits (R, 9–15 g) overripe fruits (OR, 9–15 g) and senescent fruits (SN, 9–15 g). Vegetative tissues including roots, crowns, flowers, runners and expanding leaves were also harvested. All tissues were frozen in liquid nitrogen immediately upon harvesting and stored at −80°C. *Nicotiana benthamiana* and the strawberry cultivar used for agroinfiltration (*F. × ananassa* Duch. cv. Elsanta) were grown in a plant chamber at 25°C, 10000 lux and 80% humidity, and subsequently kept in a greenhouse.

### Hormone experiments

The effects of auxin on gene regulation were examined by using two groups of ten de-achened G3 fruits for analysis in quintuplicate. The fruits in one group were covered with lanolin paste (1 mL) containing 1 mM auxin NAA dissolved in 1% (w/v) dimethyl sulfoxide, whereas those in the other, control group, were treated with a similar same paste containing no NAA. Fruits were allowed to remain on-plant throughout the experiment. Auxin treatments were repeated five times. Samples were collected and analyzed as described previously. [35]

The effects of ABA were investigated by infiltrating fruits with nordihydroguaiaretic acid (NDGA), an inhibitor of NCED enzyme activity, or silencing *FaNCED1* gene expression by RNAi. [30] These treatments are known to decrease endogenous ABA levels in ripe fruit receptacles. The fruits used (cv. Elsanta) were at an early white stage of ripening. The treatments are described in detail elsewhere. [35] Fruit pedicels were kept without media for the water stress treatment but immersed in an MS medium containing sucrose that was replaced every 2 days in the control treatment. Three pools of 10 fruits each were used in all ABA related experiments and independently processed in each experimental situation. The stress treatment, sample analysis, and ABA extraction and determination, were performed as in previous work. [29, 35] Deuterated ABA was used as internal standard. [35]

### RNA isolation and gene expression analysis by qRT-PCR

Total RNA was isolated and further purified as described elsewhere. [29] First-strand cDNA was obtained by using the iScript kit from BioRad according to the manufacturer’s instructions. Quantitative PCR was performed in triplicate with specific primers (Supplementary Table S2) on an iCycler system also from BioRad. Relative expression values were obtained with the ΔΔC_t_ method, using *interspacer 26S–18S* as housekeeping gene due to its constitutive expression in all tissues and experimental conditions. [48]

### Bioinformatic sequence analysis

Protein sequence alignment was done with EBI Clustal Omega, and InterPro for domain and functional site prediction. Physicochemical properties were determined with the pI/Mw tool in ExPASy Compute and are summarized in Supplementary Table S1. The phylogenetic tree was constructed with FigTree (http://tree.bio.ed.ac.uk/software/figtree/), using 1000 replicates for bootstrap analysis with Cello2Go (http://cello.life.nctu.edu.tw/) and Wolfpsort (http://genscript.com/psort/wolf_psort.html) for predictions of subcellular location. pI and Mw were calculated with the pI/Mw tool in ExPASy Compute (http://www.expasy.ch/tools/pi_tool.html). Major characteristics of proteins are listed in Supplementary Table S1.

### Generation of RNAi constructs and transfection of strawberry fruits by agroinfiltration

RNAi constructs were obtained by cloning a conserved 530 bp region of *FanCXE1* and a conserved 477 bp region of *FaNCED1* separately into Invitrogen’s pCR8/GW/TOPO kit, the clones being transferred to the pFRN vector (courtesy of Dr. Marten Denekamp, University of Utrecht, The Netherlands) with LR clonase, also from Invitrogen. The RNAi constructs thus obtained (pFRN-*FanCXE1* and pFRN-*FaNCED1*) were subjected to sequencing and restriction analyses prior to transformation of strawberry fruits.

For agroinfiltration, whole strawberry fruits were injected with a solution of *A. tumefaciens* strain AGL0 [49] containing either pFRN-*FanCXE1,* pFRN-*FaNCED1* or an empty pFRN (control) as described in detail elsewhere. [50] Up to 30–40 fruits from 15–25 plants were inoculated and analyzed. The extent of silencing was determined by comparing *FanCXE1* and *FaNCED1* transcript levels in pFRN-*FanCXE1* and pFRN-*FaNCED1* agroinjected fruits against those injected with empty pFRN vector 10–14 days after injection.

### Subcellular localization

The full CDS of *FanCXE1* was cloned into pDONR™221 from Invitrogen, and transferred by LR reaction to the binary vector pK7WGF2, to obtain the 35S::*GFP*::*FanCXE1* fusion construct used for localization studies. The construct was subjected to sequencing analysis prior to transformation of *Nicotiana* leaves.

Localization studies were done with *A. tumefaciens* strain GV3101. p19 protein from TBSV was used to suppress gene silencing and a plasmid containing a plasma membrane marker (PM-rk CD3–1007) tagged to mCherryFP for colocalization. [51] Cells were harvested and resuspended in an infiltration buffer containing 10 mM MgSO_4_, 10 mM MES and 1 mM acetosyringone (OD_600_ 0.5). Pairwise volumes of the cells carrying pK7WGF2-*FanCXE1*, the plasma membrane marker and the empty pK7WGF2 constructs were mixed with cells containing p19 vector and allowed to stand at room temperature in the dark for 2–4 h. Abaxial surfaces were injected by using a syringe with no needle. After 4 days, leaves were examined under an LSM 880 epifluorescence microscope from Carl Zeiss, Inc. (Dresden, Germany).

### Cloning of full-length cDNA in *FanCXE1* and expression of recombinant protein in *Escherichia coli*

The full-length cDNA of *FanCXE1* was amplified from total cDNA in strawberry red fruit by using designed primers matching the initial and final transcription zones of the gene. The oligonucleotides introduced an EcoRI and an XhoI restriction site at the 5′ and 3′ end respectively, of the *FanCXE1* PCR fragment, and were used to clone the fragment into pGEM-T-easy. The FanCXE1-GST tag was semipurified with vector pGEX-4 T-1 from Amersham. The empty pGEX-4 T-1 vector was used as control in all tests. Total protein was extracted from an overnight *E. coli* (Blb21 Gold DE3 strain, Stratagene) culture induced with 1 mM IPTG at 16°C. After 12 hours of growth, cells were harvested by centrifugation and the pellet thus obtained was frozen at −80°C for 15 min, after which it was resuspended in 10 ml of 1× ice-cold wash buffer (4.3 mM Na2HPO4·7H2O, 1.47 mM KH2PO4, 0.137 M NaCl and 2.7 mM KCl, pH 7.3) and sonicated on ice at least three times for 30 s with 10% energy by using a Sonopuls GM 2017 sonicator from Bandelin. Sonicated cells were centrifuged and 10 000 g at 4°C for 20 min and the soluble protein fraction (supernatant) was incubated at 4°C in 15 mL Telos TelosTM SPE filtration columns packed with GST-sepharose from Novogen for at least 60 min. Protein attached to sepharose was washed three times with 8 mL of 1× ice-cold wash buffer at 4°C. Finally, sepharose-bound protein was released by incubation for 5 min in 300 μL of 1× elution buffer (50 mM Tris–HCl, 1 mM glutathione, pH 8) at room temperature. The eluate was quantified with Bradford’s method (Bradford, 1976) and analyzed by SDS–PAGE (12% Progel TRIS-Glycine, 1.0 mm, Anamed).

### Enzyme assay

FanCXE1 activity was assessed by following the protocol of Gomori [52] as modified by van Asperen. [53] Briefly, an amount of 50–100 μg of semipurified enzyme from three different extracts was incubated with a 0.3 mM concentration of substrate at 27°C for 15 min, after which the reaction was stopped by adding DBLS reagent and developed color measured spectrophotometrically at 600 nm. Protein concentration was determined by using Bradford’s method. [54]

## Kinetic studies

### Influence of time, enzyme concentration and substrate specificity

The influence of time was evaluated by incubating FanCXE1 semipurified protein with 0.3 mM α-naphthyl acetate at 27°C for 10–60 min. For enzyme concentrations, 25 to 150 μg of protein were used in the reaction incubating with α-naphthyl butyrate and β-naphthyl hexanoate as substrate. In all cases, the reaction was stopped as described above and the amount of 1-naphthol released at each incubation time determined from a standard graph.

### *K_m_ and V*_max_

Purified carboxylesterase FanCXE1 was incubated with variable concentrations of the substrates α-naphthyl acetate, α-naphthyl butyrate and β-naphthyl hexanoate for 20 min, using the optimum temperature and pH, and following the above-described procedure. The product, 1-naphthol, was determined colorimetrically.

### Volatile extraction and identification

Volatiles were determined by transferring approximately 300 mg of lyophilized receptacle to 20 mL glass vials as described elsewhere. [55] For data acquisition vials were incubated at 50°C with agitation at 250 rpm in an autosampler. The SPME holder was inserted 24 mm into the vial and kept there for 20 min (extraction time), after which the sample was injected in the pulsed splitless mode, using helium at 1 mL/min as carrier gas. The injection temperature was set at 250°C and helium at the same temperature was passed through the fiber for 5 min. Chromatography was done with a 60 m DB-624 capillary column. The temperature program was as follows: 2 min at 40°C, then a 10°C/ min ramp to 260°C, which was held for 10 min. Samples were cooled down as rapidly as the instrument allowed. The mass range spanned was *m*/*z* 30–300 and data were acquired at a rate of 2 scans/s. Individual compounds were identified by using GMD or NIST standards as described elsewhere. [55]

## Statistical analysis

All statistical analyses were performed with the software GraphPad. Relative metabolite concentrations and the results of the Pareto method were processed with the software MetaboAnalyst v 4.0.

## Acknowledgements

This work was funded by the Spanish Ministry of Science and Innovation (Projects AGL2014-55784-C2-2-R and AGL2017-86531-C2-2-R); MINECO (Ramon y Cajal Project RYC-2014-15111); and the Andalusian Regional Government (Plan PAIDI2020, where FJMH is granted as postdoctoral fellow). The funders played no role in the study design, data analysis and interpretation, or manuscript writing, but simply provided funding. SA and ARF acknowledge additional funding by the European Union’s Horizon 2020 Research and Innovation Programme in the framework of the Project PlantaSyst (SGA-CSA no. 664621 and no. 739582 under FPA no. 664620).

## Data availability

The authors confirm that all data referred to here are available in the body of this article and its supplementary materials.

## Conflict of interest

The authors declare no competing interests.

## Supplementary data

Supplementary data is available at *Horticulture Research Journal* online.

## Supplementary Material

suppl_data_uhac095
