# Supplementary material for: Strawberry fruit FanCXE1 carboxylesterase is involved in the catabolism of volatile esters during the ripening process
Source: Hortic Res. 2022 Apr 22;9:uhac095. doi: 10.1093/hr/uhac095 (PMC9249579; doi:10.1093/hr/uhac095)
Supplement: suppl_data_uhac095 [file suppl_data_uhac095.zip › Martinez-Rivas et al HortRes Supporting information 31032022.pdf]

Strawberry fruit FanCXE1 carboxylesterase is involved in the catabolism of volatile esters during the ripening process.

Félix J Martínez-Rivas, Rosario Blanco-Portales, Enriqueta Moyano, Saleh Alseekh, José L Caballero, Wilfried Schwab, Alisdair R Fernie, Juan Muñoz-Blanco, Francisco J Molina-Hidalgo.

Supplementary information

Figure S1. Predicted domain present in the FanCXE1 amino acid sequence as determined with InterProScan. An alpha/beta hydrolase domain is present in the sequence.

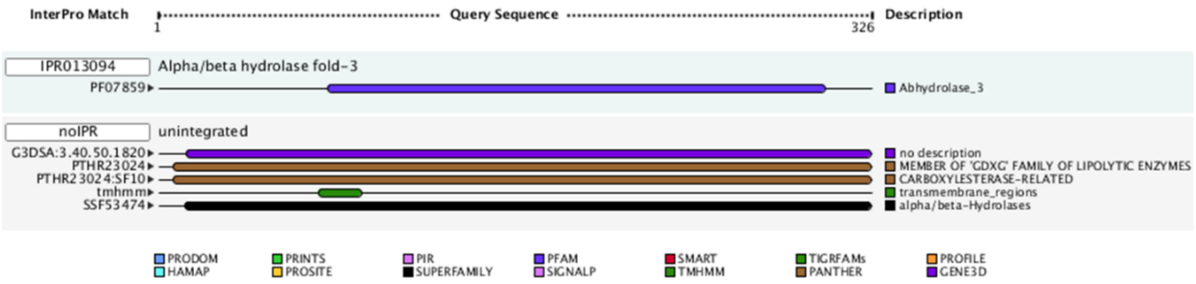

Figure S2. Phylogenetic tree of plant CXE family members. Clade III is highlighted in red. CXE protein sequences predicted from cDNA sequences. Abbreviations for species, carboxylesterase names and their GenBank accessions are as follows:

Species	Gene used
Peach (*Prunus persica*)	PpCXE1: Prupe.8G121900.1
	PpCXE2: Prupe.8G120800.1
	PpCXE3: Prupe.8G121100.1
	PpCXE4: Prupe.8G120900.1
	PpCXE5: Prupe.8G121000.1
	PpCXE6: Prupe.8G121200.1
	PpCXE7: Prupe.8G121300.1
	PpCXE8: Prupe.8G121400.1
	PpCXE9: Prupe.8G121500.1
	PpCXE10: Prupe.8G121700.1
	PpCXE11: Prupe.8G122000.1
	PpCXE12: Prupe.1G155100.1
	PpCXE13: Prupe.1G155200.1
	PpCXE14: Prupe.1G439300.1
	PpCXE16: Prupe.1G237400.1
	PpCXE17: Prupe.3G299300.1
	PpCXE18: Prupe.2G149600.1
	PpCXE19: Prupe.2G286500.1
	PpCXE20: Prupe.2G286600.1
	PpCXE21: Prupe.2G286700.1
	PpCXE22: Prupe.4G252600.1
	PpCXE23: Prupe.6G332800.1
	PpCXE24: Prupe.8G004100.1
	PpCXE25: Prupe.8G090400.1
	PpCXE26: Prupe.8G249800.1
	PpCXE27: Prupe.6G149100.1
	PpCXE28: Prupe.7G089000.1
	PpCXE29: Prupe.1G307700.1
	PpCXE30: Prupe.3G238500.1
	PpCXE31: Prupe.2G044700.1
	PpCXE32: Prupe.2G045000.1
	PpCXE33: Prupe.2G045300.1
Fragaria × ananassa	Fxa2Ag100618.1
	Fxa2Ag100622.1
	Fxa2Ag100623.1
	Fxa2Ag100626.1
	Fxa2Ag101983.1
	Fxa2Ag102925.1
	Fxa2Ag103391.1
	Fxa2Ag103537.1
	Fxa2Ag103537.2
	Fxa2Ag103537.3
	Fxa2Bg200517.1
	Fxa2Bg200521.1
	Fxa2Bg200526.1
	Fxa2Bg201719.1
	Fxa2Bg201830.1
	Fxa2Bg203180.1
	Fxa2Cg200522.1
	Fxa2Cg200522.2
	Fxa2Cg200522.3
	Fxa2Cg200678.1
	Fxa2Cg201689.1
	Fxa2Dg200607.1
	Fxa2Dg200608.1
	Fxa2Dg200613.1
	Fxa2Dg200829.1
	Fxa2Dg202547.1
	Fxa2Dg202996.1

	Fxa2Dg203106.3<br>Fxa2Dg203106.1<br>Fxa2Dg203106.2<br>Fxa3Ag100103.1<br>Fxa3Bg200106.1<br>Fxa3Cg100097.1<br>Fxa3Cg103414.1<br>Fxa3Dg200091.1<br>Fxa4Ag100330.1<br>Fxa4Ag100330.2<br>Fxa4Ag101504.1<br>Fxa4Ag102404.1<br>Fxa4Ag102678.1<br>Fxa4Ag102736.1<br>Fxa4Ag102736.2<br>Fxa4Bg100331.1<br>Fxa4Bg100331.2<br>Fxa4Bg101498.1<br>Fxa4Bg102360.1<br>Fxa4Bg102613.1<br>Fxa4Bg102661.1<br>Fxa4Bg102661.2<br>Fxa4Cg201288.1<br>Fxa4Cg202054.1<br>Fxa4Cg202359.1<br>Fxa4Cg202411.1<br>Fxa4Cg202411.2<br>Fxa4Dg100302.1<br>Fxa4Dg100302.2<br>Fxa4Dg101189.1<br>Fxa4Dg101191.1<br>Fxa4Dg101192.1<br>Fxa4Dg101898.1<br>Fxa4Dg102135.1<br>Fxa4Dg102191.1<br>Fxa4Dg102191.2<br>Fxa6Ag100417.1<br>Fxa6Ag102066.1<br>Fxa6Ag104292.1<br>Fxa6Ag104771.1<br>Fxa6Ag104772.1<br>Fxa6Ag104775.3<br>Fxa6Ag104775.2<br>Fxa6Ag104775.5<br>Fxa6Ag104775.4<br>Fxa6Ag104775.1<br>Fxa6Ag104775.7<br>Fxa6Ag104775.6<br>Fxa6Ag104775.9<br>Fxa6Ag104775.8<br>Fxa6Ag104989.1<br>Fxa6Bg100390.1<br>Fxa6Bg104331.1<br>Fxa6Bg104331.2<br>Fxa6Bg104333.5<br>Fxa6Bg104333.4<br>Fxa6Bg104333.3<br>Fxa6Bg104333.2<br>Fxa6Bg104333.1<br>Fxa6Bg104333.8<br>Fxa6Bg104333.7<br>Fxa6Bg104333.6<br>Fxa6Bg104333.11<br>Fxa6Bg104333.10<br>Fxa6Bg104333.9<br>Fxa6Bg104505.1

	Fxa6Cg100395.1<br>Fxa6Cg104252.1<br>Fxa6Cg104452.1<br>Fxa6Dg100335.1<br>Fxa6Dg101780.1<br>Fxa6Dg103744.1<br>Fxa6Dg104329.1<br>Fxa7Ag200605.1<br>Fxa7Ag200605.2<br>Fxa7Ag200605.3<br>Fxa7Ag201290.1<br>Fxa7Bg200641.1<br>Fxa7Bg200641.2<br>Fxa7Bg202677.1<br>Fxa7Cg101192.1<br>Fxa7Cg102649.1<br>Fxa7Dg102456.1
Fragaria vesca	FvH4_1g03850.t3<br>FvH4_1g03850.t1<br>FvH4_1g03850.t4<br>FvH4_1g03850.t2<br>FvH4_2g06400.t1<br>FvH4_2g06430.t1<br>FvH4_2g06450.t1<br>FvH4_2g06480.t1<br>FvH4_2g06490.t1<br>FvH4_2g06520.t1<br>FvH4_2g06530.t1<br>FvH4_2g06540.t1<br>FvH4_2g06560.t1<br>FvH4_2g06620.t1<br>FvH4_2g18461.t1<br>FvH4_2g19350.t1<br>FvH4_2g29190.t1<br>FvH4_2g34680.t1<br>FvH4_2g34730.t1<br>FvH4_3g01110.t1<br>FvH4_4g02821.t1<br>FvH4_4g15000.t1<br>FvH4_4g15010.t1<br>FvH4_4g15020.t1<br>FvH4_4g24810.t1<br>FvH4_4g27710.t1<br>FvH4_4g28400.t1<br>FvH4_4g28400.t3<br>FvH4_4g28400.t2<br>FvH4_4g28400.t4<br>FvH4_6g04960.t2<br>FvH4_6g04960.t3<br>FvH4_6g04960.t4<br>FvH4_6g04960.t1<br>FvH4_6g21100.t1<br>FvH4_6g21100.t2<br>FvH4_6g45240.t1<br>FvH4_6g45510.t1<br>FvH4_6g45510.t4<br>FvH4_6g45510.t5<br>FvH4_6g45510.t6<br>FvH4_6g45510.t3<br>FvH4_6g45510.t2<br>FvH4_6g45510.t7<br>FvH4_6g50160.t1<br>FvH4_6g50160.t3<br>FvH4_6g50160.t2<br>FvH4_6g50162.t1<br>FvH4_6g52540.t1

	FvH4_6g52540.t2<br>FvH4_7g12500.t1<br>FvH4_7g28650.t1
Apple (*Malus domestica*)	MdCXE1: ES789959;<br>MdCXE2: ES789969;<br>MdCXE3: ES789964;<br>MdCXE4: ES789967;<br>MdCXE5: ES789968;<br>MdCXE6: ES789979;<br>MdCXE7: ES789974;<br>MdCXE8: ES789984;<br>MdCXE9: ES789966;<br>MdCXE10: ES789970;<br>MdCXE11: EG631279;<br>MdCXE12: ES790010,<br>MdCXE13: ES789965;<br>MdCXE14: EG631362;<br>MdCXE15: CN942679;<br>MdCXE16: EB106571
Arabidopsis (*Arabidopsis thaliana*)	AtCXE1: NP_173353.1;<br>AtCXE2: NP_564507.1;<br>AtCXE3: NP_175387.1;<br>AtCXE4: NP_564552.1;<br>AtCXE5: NP_175389.1;<br>AtCXE6: NP_564936.1;<br>AtCXE7: NP_178453.1;<br>AtCXE8: NP_566047.1;<br>AtCXE9: NP_182085.1;<br>AtCXE10: NP_187163.1;<br>AtCXE11: NP_180300.1;<br>AtCXE12: NP_190438.1;<br>AtCXE13: NP_190439.1;<br>AtCXE14: NP_191860.1;<br>AtCXE15: NP_196275.1;<br>AtCXE16: NP_568298.1;<br>AtCXE17: NP_197112.1;<br>AtCXE18: NP_197744.1;<br>AtCXE19: NP_198084.1;<br>AtCXE20: NP_201024.
Grape (*Vitis vinifera)*	VvBIG8.1: AF487826.1
Kiwifruit (*Actinidia eriantha)*	AeCXE1: DQ279914
Licorice (*Glycyrrhiza echinate)*	GeHIDM: BAD80839.1
Pepper (*Capsicum annuum*)	CaPepEST, AAF77578.1
Pine (*Pinus radiate)*	PrMC3: AAD04946.2
Soybean (*Glycine max*)	GmHIDH: BAD80840.1
Tobacco (*Nicotiana tabacum*)	hsr203j, AAF62404.1
Rice (*Oryza sativa)*	GID1: AAV59435.1).

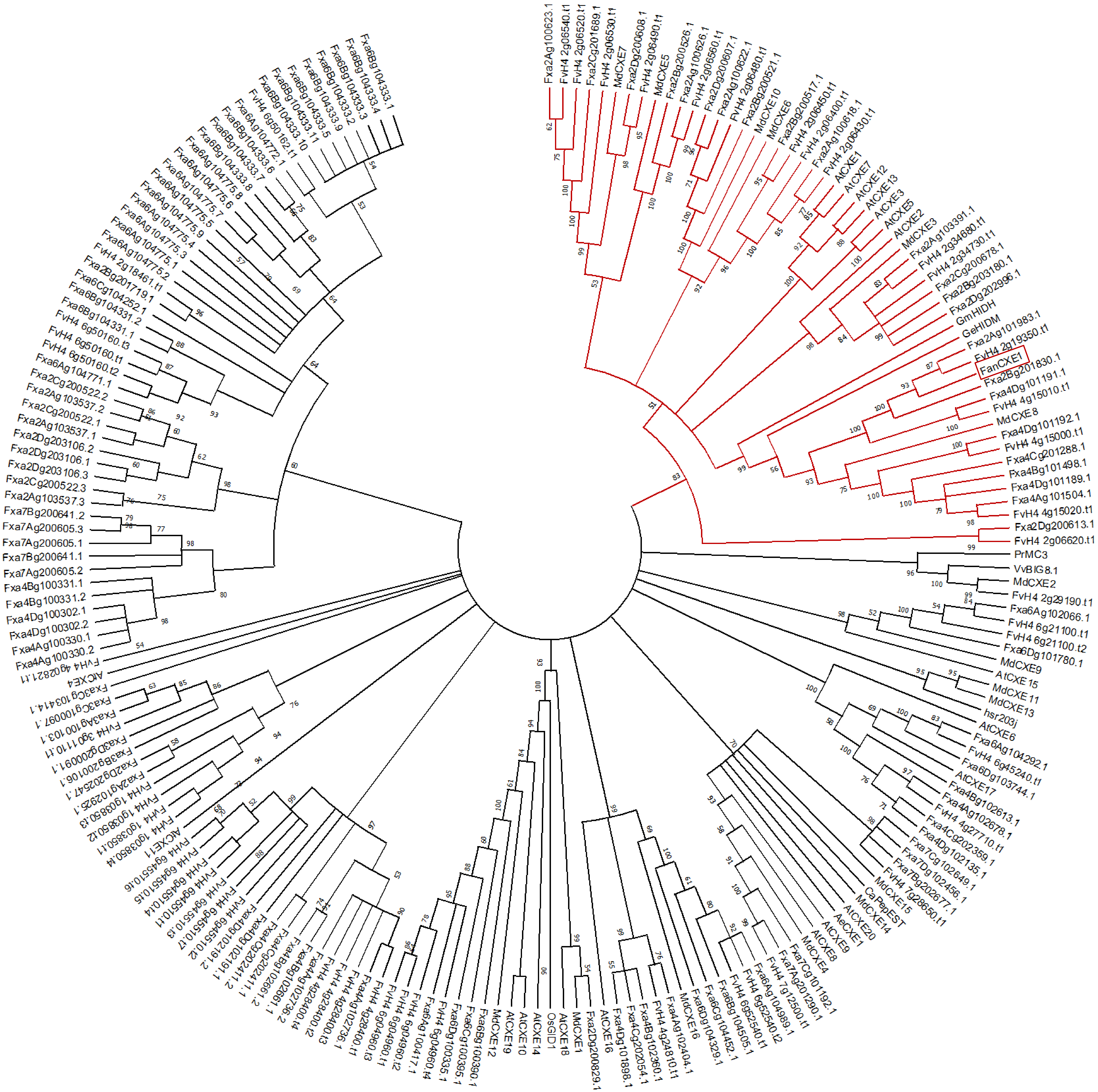

Fxa2Ag100623.1
FvH4 2g06540.t1
FvH4 2g06520.t1
Fxa2Cg201689.1
FvH4 2g06530.t1
MdCXE7
Fxa2Dg200608.1
FvH4 2g06490.t1
MdCXE5
Fxa2Bg200526.1
Fxa2Ag100626.1
FvH4 2g06560.t1
Fxa2Dg200607.1
Fxa2Ag100622.1
FvH4 2g06480.t1
Fxa2Bg200521.1
MdCXE10
MdCXE6
Fxa2Bg200517.1
FvH4 2g06450.t1
FvH4 2g06400.t1
Fxa2Ag100618.1
FvH4 2g06430.t1
AtCXE1
AtCXE7
AtCXE12
AtCXE13
AtCXE3
AtCXE5
AtCXE2
MdCXE3
Fxa2Ag103391.1
FvH4 2g34680.t1
FvH4 2g34730.t1
Fxa2Cg200678.1
Fxa2Bg203180.1
Fxa2Dg202996.1
GmHIDH
GeHIDH
Fxa2Ag101983.1
FvH4 2g19350.t1
FanCXE1
Fxa2Bg201830.1
Fxa4Dg101191.1
FvH4 4g15010.t1
MdCXE8
Fxa4Dg101192.1
FvH4 4g15000.t1
Fxa4Cg201288.1
Fxa4Bg101498.1
Fxa4Dg101189.1
Fxa4Ag101504.1
FvH4 4g15020.t1
Fxa2Dg200613.1
FvH4 2g06620.t1
PrMC3
VvBIG8.1
MdCXE2
FvH4 2g29190.t1
Fxa6Ag102066.1
FvH4 6g21100.t1
FvH4 6g21100.t2
Fxa6Dg101780.1
MdCXE9
AtCXE15
MdCXE11
MdCXE13
hsr203j
AtCXE6
Fxa6Ag104292.1
FvH4 6g45240.t1
Fxa6Dg103744.1
AtCXE17
Fxa4Bg102613.1
Fxa4Ag102678.1
FvH4 4g27710.t1
Fxa4Cg202359.1
Fxa4Dg102135.1
Fxa7Cg102649.1
Fxa7Dg102456.1
Fxa7Bg202677.1
FvH4 7g28650.t1
MdCXE15
CaPepEST
MdCXE14
AtCXE20
AeCXE1
AtCXE9
AtCXE8
MdCXE4
Fxa7Cg101192.1
Fxa7Ag201290.1
FvH4 7g12500.t1
Fxa6Ag104989.1
FvH4 6g52540.t2
FvH4 6g52540.t1
Fxa6Bg104505.1
Fxa6Cg104452.1
Fxa6Dg104329.1
MdCXE16
Fxa4Ag102404.1
FvH4 4g24810.t1
Fxa4Bg102360.1
Fxa4Cg202054.1
Fxa4Dg101898.1
AtCXE16
Fxa2Dg200829.1
MdCXE1
AtCXE18
OsGID1
AtCXE14
AtCXE10
AtCXE19
MdCXE12
Fxa6Bg100390.1
Fxa6Cg100395.1
Fxa6Dg100335.1
FvH4 6g04960.t4
Fxa6Ag100417.1
FvH4 6g04960.t2
FvH4 6g04960.t1
FvH4 6g04960.t3
FvH4 4g28400.t1
Fxa4Ag102736.1
FvH4 4g28400.t3
FvH4 4g28400.t2
FvH4 4g28400.t4
Fxa4Ag102736.2
Fxa4Bg102661.1
Fxa4Bg102661.2
Fxa4Cg202411.1
Fxa4Cg202411.2
Fxa4Dg102191.1
Fxa4Dg102191.2
FvH4 6g45510.t2
FvH4 6g45510.t7
FvH4 6g45510.t3
FvH4 6g45510.t1
FvH4 6g45510.t4
FvH4 6g45510.t5
FvH4 6g45510.t6
AtCXE11
FvH4 1g03850.t4
FvH4 1g03850.t1
FvH4 1g03850.t2
FvH4 1g03850.t3
Fxa1Ag102925.1
Fxa2Dg202547.1
Fxa2Bg200106.1
Fxa3Dg200091.1
FvH4 3g01110.t1
Fxa3Ag100103.1
Fxa3Cg100097.1
Fxa3Cg103414.1
AtCXE4
FvH4 4g02821.t1
Fxa4Ag100330.2
Fxa4Ag100330.1
Fxa4Dg100302.2
Fxa4Dg100302.1
Fxa4Bg100331.2
Fxa4Bg100331.1
Fxa7Ag200605.2
Fxa7Bg200641.1
Fxa7Ag200605.1
Fxa7Ag200605.3
Fxa7Bg200641.2
Fxa2Ag103537.3
Fxa2Cg200522.3
Fxa2Dg203106.3
Fxa2Dg203106.1
Fxa2Dg203106.2
Fxa2Ag103537.1
Fxa2Cg200522.1
Fxa2Ag103537.2
Fxa2Cg200522.2
Fxa6Ag104771.1
FvH4 6g50160.t2
FvH4 6g50160.t1
FvH4 6g50160.t3
Fxa6Bg104331.1
Fxa6Bg104331.2
Fxa6Cg104252.1
Fxa2Bg201719.1
FvH4 2g18461.t1
Fxa6Ag104775.2
Fxa6Ag104775.1
Fxa6Ag104775.3
Fxa6Ag104775.4
Fxa6Ag104775.5
Fxa6Ag104775.9
Fxa6Ag104775.7
Fxa6Ag104775.6
Fxa6Ag104775.8
Fxa6Bg104333.8
Fxa6Bg104333.6
Fxa6Bg104333.7
Fxa6Ag104772.1
FvH4 6g50162.t1
Fxa6Bg104333.10
Fxa6Bg104333.11
Fxa6Bg104333.5
Fxa6Bg104333.9
Fxa6Bg104333.2
Fxa6Bg104333.3
Fxa6Bg104333.4
Fxa6Bg104333.1

Figure S3. Alignment of the amino acid sequences of FanCXE1, MdCXE8, PpCXE12, PpCXE13, GeHIDM and GmHIDH. The FanCXE1 α/β hydrolase predicted domain (InterProScan 5) is highlighted in red. Putative residues constituting the catalytic triad (Ser/Thr, Asp, and His) are denoted with a dot (●). Also, the GXS/TXG sequence and the oxyanion hole (Gly-Gly) motifs are underlined.

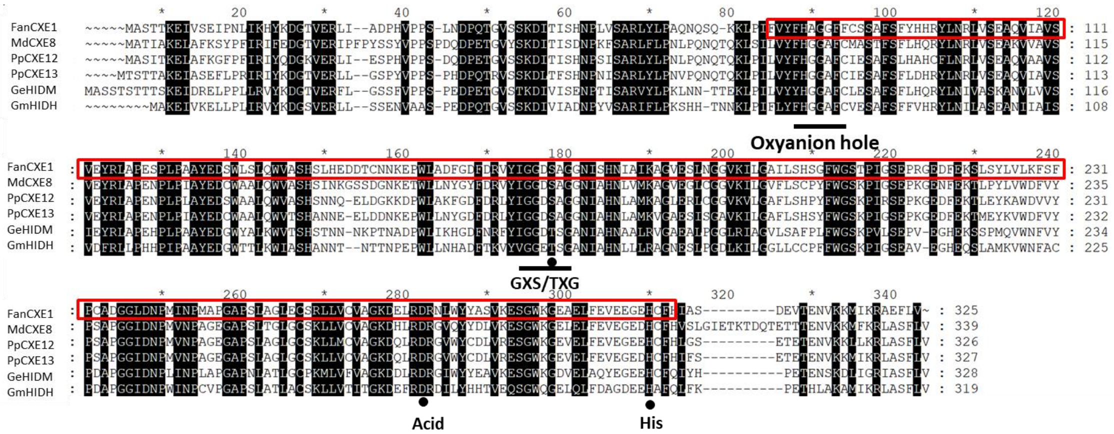

Figure S4. Overexpression of FanCXE1-GST in *E. coli* transformed with the pGEX-4T-1-CXE2 vector. The recombinant protein had MW = 64 kDa. The vector pGEX-4T-1 (Amersham) was used for expression of recombinant FanCXE1 protein in *E. coli* cells (Blb21 Gold DE3 strain, Stratagene). F.1, F.2 and F.3 correspond to three independent induction and purification runs. Staining was done with Coomasie Blue.

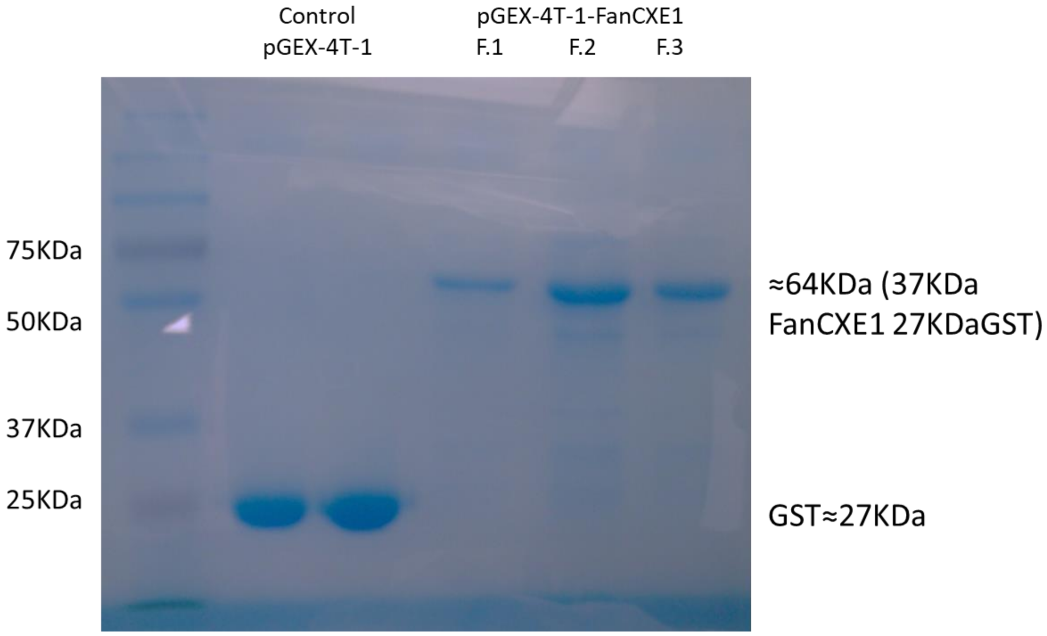

Figure S5. (A) Activity of strawberry recombinant protein, FanCXE1, at different temperatures in a 0.3 mM substrate solution in 0.05 M sodium phosphate buffer at pH 7.0. The effect of temperature on activity in the purified esterase was examined over the range 10–50 °C. The enzyme was diluted and incubated with the substrate α-naphthyl acetate at different temperatures for 60 min. The amount of 1-naphthol released was measured colorimetrically. (B) Activity of strawberry recombinant protein, FanCXE1, at different pH values in a 0.3 mM substrate solution at 27 °C. The effect of pH on activity in the purified esterase was examined by using the following buffers: (1) 0.05 M sodium citrate (pH 3.0–6.0), (2) 0.05 M sodium phosphate (pH 6.0–8.0) and (3) 0.05 M Tris–HCl (pH 8.0–10.0). The catalytic activity of the enzyme in the previous buffers was determined with α-naphthyl acetate as substrate. The reaction was conducted at 27 °C for 15 min and 1-naphthol formed was determined colorimetrically. In both cases, controls were prepared by using GST protein to assess nonenzymatic hydrolysis of the substrate. Error bars represent standard errors of the means as calculated from a minimum of three replicates over three experiments.

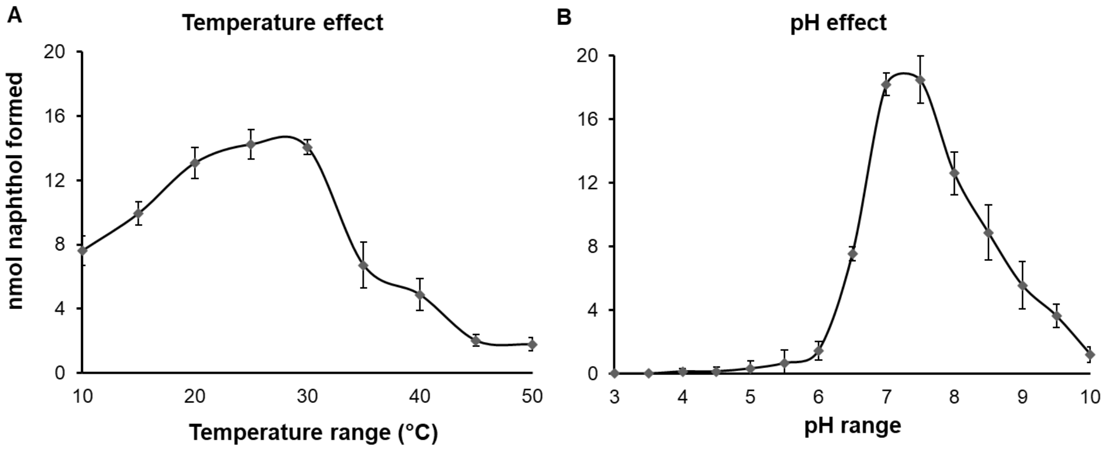

Figure S6. Analysis by qRT-PCR of *FanCXE1* gene expression in transgenic *FanCXE1*-RNAi strawberry fruits (*F.* × *ananassa* cv. Elsanta) agroinfiltrated with empty pFRN vector (control) and the pFRN-*FanCXE1* construct. Statistical significance with respect to the reference sample was determined with Student's *t*-test in all experiments. (*) $p < 0.01$. Pool 1 fruits 1, 2, 5; pool 2 fruits 12, 16, 22; pool 3 fruits 29, 30, 35.

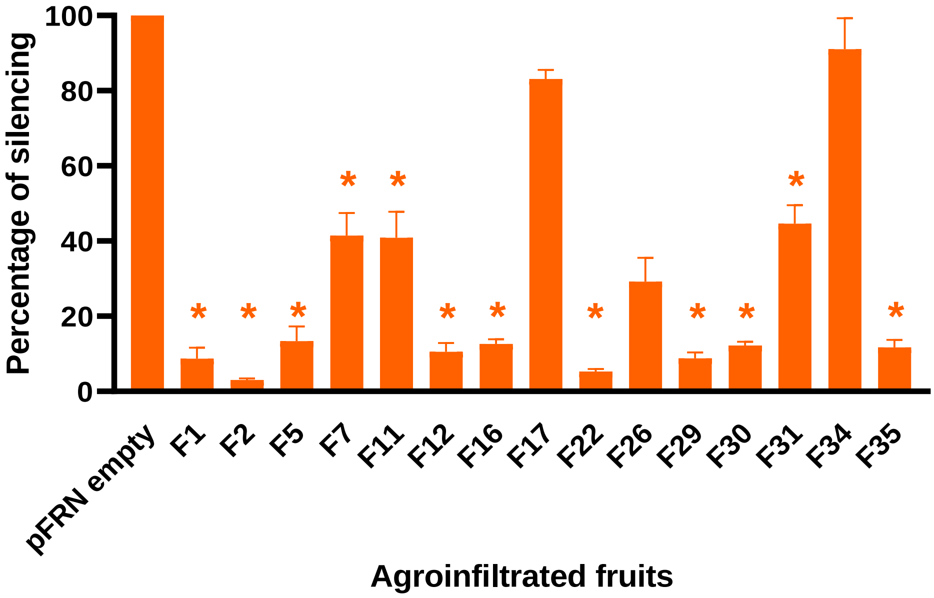

Table S1. Predicted properties of different carboxylesterases characterized in plants and of the intracellular locus of the proteins on two different websites.

	FanCXE1	MdCXE8	GeHIDM	PpCXE1	GmHIDH
**NCBI Acc. No**	XP_004290227.1	ES789984	BAD80839.1	ONH91534.1	BAD80840.1
**Protein size**	325	339	328	315	319
**pI**	5,29	5,46	5,41	6,15	5,75
**MW (Da)**	35789,38	37528,71	36060,81	34469,3	35138,09
**Prediction of intracellular localization of the proteins**					
**Cello2go**	cytoplasm	chloroplast	cytoplasm	cytoplasm	cytoplasm
**WolfPSORT**	cytoplasm	chloroplast	cytoplasm	cytoplasm	cytoplasm

Table S2. Primer sequences used in this work. Fw forward; Rv reverse. Up upper; Low lower.

Gene	Purpose	Primer	Sequence 5'-3'
FanCXE1	FL sequence	FanCXE1FL-Fw	gtagtagaattcATGGCTTCCACGACCAAAGAAATCGTCT
		FanCXE1FL-Rv	tactacctcgagCTAGACTAGAAAATCTGCCATCCGTTTG
	GFP Fusion	CXE1-loc-up	GGGGACAAGTTTGTACAAAAAAGCAGGCTTAACAATGGCTTCCACGACCAAAGAAATCGTCT
		CXE1-loc-low	GGGGACCACTTTGTACAAGAAAGCTGGGTCCTAGACTAGAAAATCTGCCATCCGTT
	qRT-PCR	CXE1-Up	CCCATTCGGGTTTCTGGGGA
		CXE1-Low	TCATCCTTGCCAGCGACACA
	RNAi construct	CXE1-RNAiUp	TACCACCACCGCTACCTCAA
		CXE1-RNAiLow	TCCTTGCCAGCGACACAAAC
FaNCED1	qRT-PCR	FaNCED1-up	GAGCTTGAAGAGTGTGTTGTCCGAAA
		FaNCED1-low	CGGTTCAGCAATGGCGAGATAAGCATA
Interspacer 26S-18S	qRT-PCR	413fw	ACCGTTGATTCGCACAATTGGTCATCG
		413rv	TACTGCGGGTCGGCAATCGGACG
